# Knockdown long noncoding RNA nuclear paraspeckle assembly transcript 1 suppresses colorectal cancer through modulating miR‐193a‐3p/KRAS

**DOI:** 10.1002/cam4.1798

**Published:** 2018-12-21

**Authors:** Zhouting Zhu, Shangce Du, Kai Yin, Shichao Ai, Mengchao Yu, Yanqing Liu, Yan Shen, Minghui Liu, Ruihua Jiao, Xi Chen, Wenxian Guan

**Affiliations:** ^1^ Department of General Surgery Affiliated Drum Tower Hospital of Nanjing University Medical School Nanjing China; ^2^ State Key Laboratory of Pharmaceutical Biotechnology, NJU Advanced Institute for Life Sciences (NAILS), Jiangsu Engineering Research Center for MicroRNA Biology and Biotechnology, School of Life Sciences Nanjing University Nanjing China; ^3^ Department of General Surgery Drum Tower Clinical Medical College of Nanjing Medical University Nanjing China; ^4^ Department of General Surgery Taixing Hospital Affiliated to Yangzhou University Taixing China

## Abstract

The nuclear paraspeckle assembly transcript 1 (abbreviated as NEAT1), a nuclear sufficient long noncoding RNA (abbreviated as lncRNA), has aroused a rising concern in recent years. As uncovered by reports, the increase in NEAT1 is related to the deteriorated prognosis of lung cancer, breast cancer, hepatocellular cancer, and colorectal cancer (abbreviated as CRC). Thus far, the mechanism of NEAT1 has not been elucidated by the existing researches. The impact of knockdown of both NEAT1 and its predicted downstream miR‐193a‐3p in CRC cells was examined here to delve into their interactions and mechanisms. Additionally, the target of miR‐193a‐3p, Kirsten rat sarcoma viral oncogene homolog (abbreviated as KRAS), was also predicted by bioinformatics algorithms. Small interfering RNA and antisense oligonucleotides that inhibit NEAT1, as well as overexpression or knockdown of miR‐193a‐3p, were adequately drawn upon to confirm that NEAT1 serves as a miR‐193a‐3p sponge or competing endogenous RNA, to impact miR‐193a‐3p's further functions, including modulating KRAS proteins, both in vitro and in vivo. Generally, lncRNA NEAT1/hsa‐miR‐193a‐3p/KRAS axis was substantiated in CRC cells and could provide novel insight into both diagnostic and therapeutic advancement in CRC.

## 1 INTRODUCTION

Colorectal cancer (abbreviated as CRC), the third most common cancer type and the third most dominating cause of cancer‐related deaths in the United States,^1^ has aroused rising concern because of its high morbidity and mortality. The formation of CRC is mediated jointly by genetic alterations and nongenetic alterations. Nongenetic factors contain age, gender, high fat intake, obesity, alcohol, and deficiency of physical exercise.^2^ Kirsten rat sarcoma viral oncogene homolog (abbreviated as KRAS) signaling pathway is one of the most commonly activated pathways among genetic alterations, aggravating the occurrence and progression of intestinal neoplasms.^3^ The KRAS gene, situating at 12p 12.1, encodes KRAS protein pertaining to the small GTPase superfamily. Anomalous KRAS protein activation affects the normal RAS/PI3K/AKT signaling, as well as cell proliferation, invasion, and apoptosis.^4^ Accordingly, KRAS pertains to an oncogene family to cause cancerous change. Aberrant level and somatic activating mutations in KRAS gene are pervasively found in human cancers, inclusive of CRC,^5^ pancreatic cancer,^6^ gastric cancer,^7^ breast cancer,^8^ and lung cancer.^9^ In this regard, targeting KRAS gene is promising to treat cancers like CRC.

Long noncoding RNA (abbreviated as lncRNAs) are transcripts with in excess of 200 nucleotides (nt) length. lncRNAs are classified into nuclear lncRNAs^10^ and cytoplasmic lncRNAs.^11^ Generally, lncRNAs work as modulators in cellular processes. Aberrant expression of lncRNAs is believed to lead to pathological disorders and even cancer.^12^ For example, lncRNA HOTAIR is highly expressed in breast cancer and targets chromatin repressor Polycomb proteins to specific genomic loci, which could promote cancer metastasis^13^; MALAT1 is associated with tumor cell proliferation, invasion, metastasis in lung cancer, breast cancer, bladder cancer, etc^14^; H19, embeds with miR‐675, significantly overexpresses in various types of tumors and regulates downstream targets to promote cell proliferation and inhibits cell apoptosis in oncogenetic developments.^15^

Nuclear paraspeckle assembly transcript 1 (abbreviated as NEAT1), located on chromosome 11 (11q13.1), is one of nuclear lncRNAs and has been identified to play crucial roles in various cancers. NEAT1 has 2 isoforms: 3.7knt NEAT1_1 with poly(A) tail and 23knt NEAT1_2, both of which are crucial components of the paraspeckles.^10^ NEAT1 is found upregulated in most cancers, and higher expression of NEAT1 relates to poor prognosis in cancer.^12^ For instance, NEAT1 was identified by Wu et al^16^ as a diagnostic and prognostic biomarker in colorectal cancer. Zhang et al^17^ reported NEAT1 promotes proliferation and EMT in breast cancer. Recently, lncRNAs that serve as endogenous miRNA sponges in a part of competing endogenous RNA (ceRNA) network are drawing more attentions. NEAT1‐miR‐129‐5p‐KLK7 pathway was validated by Zhang et al^18^ in papillary thyroid cancer. Increased NEAT1 content was validated as a ceRNA of miR‐218 to promote cell proliferation and invasion.^19^ Yet the mechanism of NEAT1 participating in pathological developments of cancer remains unclear due to intersecting modulating networks and pathways.

MicroRNAs (abbreviated as miRNAs) are small noncoding RNAs with 19‐22 nt length that typically decrease the stability and translation of messenger RNAs (abbreviated as mRNAs) through targeting the 3′ untranslated region (abbreviated as 3′UTR) of mRNAs.^20^ miRNAs are critical for cell functions, for example cell cycle, proliferation, and invasion. Dysregulation of miRNA expression can cause pathogeneses.^21^ For instance, miR‐17‐92 cluster could play critical role in suppressing G1/5 cell cycle checkpoint and increasing the uncontrolled proliferation of the cancer cells.^22^ The exosome shuttled miR‐223 promoted the invasiveness of breast cancer cells.^23^ Among all the miRNAs, miR‐193a‐3p is one of the most noteworthiness, with downregulated expression reported in NSCLC,^24^ prostate cancer,^25^ breast cancer,^26^ colorectal cancer,^27^ head and neck squamous cell carcinoma,^28^ and myeloid leukemia.^29^ Deficiency of miR‐193a‐3p's carcinogenic impact arose from miR‐193a‐3p's suppression of c‐Kit^29^ and PTEN/PI3K signaling pathway in acute myeloid leukemia,^29^ of ERBB4 in lung cancer,^30^ of KRAS in diverse tumor cells and metastasis,^31^ of GRB7 and MAPK/ERK pathways in ovarian cancer,^32^ etc. In this study, we first found CRC tissues with remarkably higher NEAT1 content and then adequately applied three bioinformatics algorithms to screen out the potential NEAT1 ceRNA target, miR‐193a‐3p. Afterward, the downstream target of miR‐193a‐3p, KRAS, was studied and validated. Lastly, the indirect control of NEAT1/KRAS was confirmed with small interfering RNA and antisense oligonucleotides both in vitro and in vivo, providing novel insight into the lncRNA‐based oncological therapeutic approach to treat human colorectal cancer.

## 2 MATERIALS AND METHODS

### 2.1 Patient tissues collection

The CRC and paired normal adjacent tissues (abbreviated as NAT) were collected from patients carrying primary CRC and all the biospecimens are provided by Nanjing multicenter biobank, biobank of Nanjing Drum Tower Hospital, the Affiliated Hospital of Nanjing University Medical School, with the consent of every donor, and normalized ethnic audit has proceeded. After dissection, tissue fragments were instantly refrigerated in liquid nitrogen during the time of surgery and then stored at −80°C.

### 2.2 Cell lines and culture conditions

Shanghai Institute of Cell Biology, Chinese Academy of Sciences (Shanghai, China) offered the human CRC cell lines SW480, HT29, and Caco2. Of the three cells, SW480 and HT29 were incubated with RPMI 1640 medium, and Caco2 was incubated with DMEM medium in addition to 10% fetal bovine serum (FBS; Gibco, Carlsbad, CA, USA) and 1% penicillin/streptomycin (Gibco) under a 5% CO_2_, water‐saturated atmosphere.

Stable cell line expression or knockdown miR‐193a‐3p was established through transfecting lentivirus (GenePharma, Shanghai, China) into SW480 cells and was selected adopting puromycin. miR‐193a‐3p mimics and inhibitors in three cell lines were transiently transfected with lipofectamine 3000 (Invitrogen, Carlsbad, CA, USA).

### 2.3 Transfection of RNAi, antisense oligonucleotides, and plasmids

Transient interference of NEAT1 adopted h‐NEAT1 Smart Silencer (Ribobio, Guangzhou, China). The stable NEAT1 knockdown cell line was achieved through transfecting cholesterol modified antisense oligonucleotides NEAT1 (Ribobio). Effective NEAT1 siRNA as well as aso sequence was presented as GGGACAGACAGGGAGAGATG. Negative controls were all offered and adopted following the manufacturer's protocols.

The plasmid particularly expresses the human KRAS open reading frame (abbreviated as ORF) without miR‐193a‐3p responsive 3′‐UTR was designed and purchased from GeneCopoeia (Germantown, MD, USA). An empty plasmid was involved in negative control.

### 2.4 RNA isolation and quantitative RT‐PCR

This study extracted cells and patient tissues with RNAiso Reagent (Takara Bio, Shiga, Japan) as instructed by the manufacturer. One microgram of overall RNA was reverse‐transcribed to cDNA with oligodT (Takara) and AMV reverse transcriptase (Takara) to quantify long noncoding RNA NEAT1 and mRNA KRAS. Conditions for reaction were at 16°C for 10 minutes, 42°C for 60 minutes, and 85°C for 5 minutes. Quantitative RT‐PCR was performed with RT product, SYBR Green dye (Invitrogen), and specific primers for NEAT1 and KRAS on an Applied Biosystems 7300 Sequence Detection System (Applied Biosystems, Carlsbad, CA, USA). Sequences are presented below: NEAT1 (sense) CAGTTAGTTTATCAGTTCTCCCATCCA; NEAT1 (antisense): GTTGTTGTCGTCACCTTTCAACTCT; KRAS (sense): GACTCTGAAGATGTACCTATGGTCCTA; KRAS (antisense): CATCATCAACACCCTGTCTTGTC. GAPDH was adopted to normalize changes and the primes are GAPDH (sense): GATATTGTTGCCATCAATGAC; GAPDH (antisense): TTGATTTTGGAGGGATCTCG. The reactions were incubated at 95°C for 5 minutes, followed by 40 cycles of 95°C for 30 seconds, 60°C for 30 seconds, and 72°C for 30 seconds. Fold change was calculated with relative quantification (2^−ΔCt^).

TaqMan miRNA probes (Applied Biosystems) were also adopted to quantify miRNA expression. RNA 1 μg was totally reverse‐transcribed to cDNA with a stem‐loop RT primer (Applied Biosystems) as well as AMV reverse transcriptase (Takara). Reaction condition was as follows: 16°C for 30 minutes, 42°C for 30 minutes, and 85°C for 5 minutes. Then, quantitative real‐time PCR was also processed with the foregoing system, at 95°C for 5 minutes, followed by 40 cycles of 95°C for 30 seconds, 60°C for 30 seconds, and 72°C for 30 seconds. The miRNA level normalized to U6 was calculated with the formula 2^−ΔCt^.

### 2.5 Protein extraction and western blotting

The cell and tissues were lysed for 30 minutes on ice jointly with RIPA lysis buffer (Beyotime, Shanghai, China) and a protease inhibitor cocktail (Thermo Scientific 78440, Rockford, IL, USA). Then, these were centrifuged at 12 000 *g* at 4 °C for 20 minutes. The supernatant was preserved, followed by the utilization of BCA protein assay kit (Thermo Scientific) to produce protein concentration. Levels of protein were analyzed by western blotting through respective antibodies and normalized by same blots GAPDH. GAPDH antibody was bought from Santa Cruz Biotechnology (sc‐30; Dallas, TX, USA), whereas KRAS from Abcam (ab172949; Cambridge, UK).

### 2.6 Luciferase reporter assay

The human KRAS's 3′‐UTR or NEAT1’s seed sequence was amplified from human genomic DNA as a template, individually incorporated into the pmiR‐RB‐REPORT™ (Thermo Scientific) and verified with DNA sequencing. The binding sites with the seed region of miR‐193a‐3p were mutated both in KRAS and NEAT1 to ascertain the binding specificity. Either mutant KRAS's 3'‐UTR or NEAT1’s seed sequence was incorporated into an equivalent luciferase reporter. 293T were co‐transfected with the luciferase reporter plasmid in 1 μg, the β‐galactosidase (β‐gal) expression plasmid in 1 μg (Thermo Scientific), as well as equal 100 pmol of miR‐193a‐3p mimics, inhibitors, or negative control RNAs with Lipofectamine 3000 (Invitrogen). Efficiency was ascertained with the β‐gal plasmid. Measurements were performed 48 hours after the transfection and adopting a luciferase assay kit (Promega, Madison, WI, USA).

### 2.7 Cell proliferation assay

SW480 cells were incubated in 96‐well plates under 1 × 10^4^ cells per well density and incubated for 2 hours in 100 μL RPMI‐1640 medium with 2% FBS for the 0‐hour measurement with the Cell Counting Kit‐8 (Selleck Chemicals, Shanghai, China). Then, the cells density would be appraised every 24 hours. Eventually, five time‐points were totally achieved. All experiments were performed in triplicate.

### 2.8 Cell migration assay

Cell migration capacity of SW480 was tested with Matrigel Invasion Chambers (BD Biosciences, Sparks, MD, USA) with an 8‐μm pore‐size membrane contained. Cells were harvested 24 hours after transfection for suspension in RPMI‐1640 culture medium without FBS and then add to the upper chamber (4 × 10^4^ cells per well). In the meantime, the lower compartments were filled with 20% FBS RPMI‐1640 medium in 0.5 mL. After 24‐hour migration, cells on the lower surface of the filter membrane were mixed for 20 minutes with 4% paraformaldehyde at ambient temperature, washed two times with 1 × PBS and eventually stained for 15 minutes with 0.5% crystal violet solution. The cells on the upper surface of the filter membrane would be scraped out with a cotton swab. Lower surfaces (with migrated cells) would be imaged with a photomicroscope (40× fields per chamber; BX51; Olympus, Tokyo, Japan). All experiments were performed in triplicate.

### 2.9 Apoptosis assays

Annexin V‐FITC/propidium iodide (PI) staining assay was used to test the apoptosis of SW480. SW480 cells that incubated in 12‐well plates were transfected desired sequence to induce apoptosis. Forty‐eight hours after the transfection in FBS‐free RPMI‐1640 medium, the attached cells were collected. The harvested cells were washed with PBS twice and then re‐suspended in binding buffer (100 mmol/L HEPES, pH 7.4; 100 mmol/L NaCl; 25 mmol/L CaCl_2_). As stained with Annexin V‐FITC/PI (BD Biosciences) in dark for 20 minutes at ambient temperature, the cells were evaluated in a fluorescence‐activated cell‐sorting (abbreviated as FACS) flow cytometer (BD). All experiments were performed in triplicate.

### 2.10 Tumorigenicity assays

SW480 cells that express desired genes were attained by following methods. miR‐193a‐3p mimics, inhibitor, and the corresponding control stable expression in SW480 were attained by lentivirus vectors. Aso NEAT1 and KRAS plasmids, as well as their control, were transiently co‐transfected with lipofectamine 3000 (Invitrogen) in Opti‐MEM (Gibco) 1 day before implantation. Five hours after the transfection, Opti‐MEM was changed for RPMI‐1640 medium with 2% FBS (Gibco). Tumorigenicity was determined by injecting these SW480 cells with Matrigel (BD Biosciences) into the 5‐week‐old nude mice (2 × 10^6^ cells per mouse, 5‐7 mice per group) in their armpits. Mice were offered by the Model Animal Research Center of Nanjing University (Nanjing, China) and housed under specific pathogen‐free conditions. The animals were euthanized nearly one month after the implantation, and tumors were removed for RNA and protein extraction, hematoxylin and eosin (H&E) staining or immunohistochemical (IHC) staining. The tumor size was measured by a caliper and calculated as tumor volume = length × width^2^/2. All researches were supported by the Institutional Review Board of Nanjing University (Nanjing, China), and experiments were performed and guided by the National Institutes.

### 2.11 Statistical analysis

All the quantitative RT‐PCR, western blotting, luciferase reporter assays, proliferation assays, migration assays, and apoptosis assays represent at least three independent repetitions. The outcomes with means ± SD were all performed in triplicate. The overall statistical analysis referred to two‐tailed Student's *t* test. All data deemed different statistically were at *P* < 0.05.

## 3 RESULTS

### 3.1 CRC tissues and cells demonstrate increase of long noncoding RNA NEAT1 with decrease of miR‐193a‐3p

**Figure 1:**
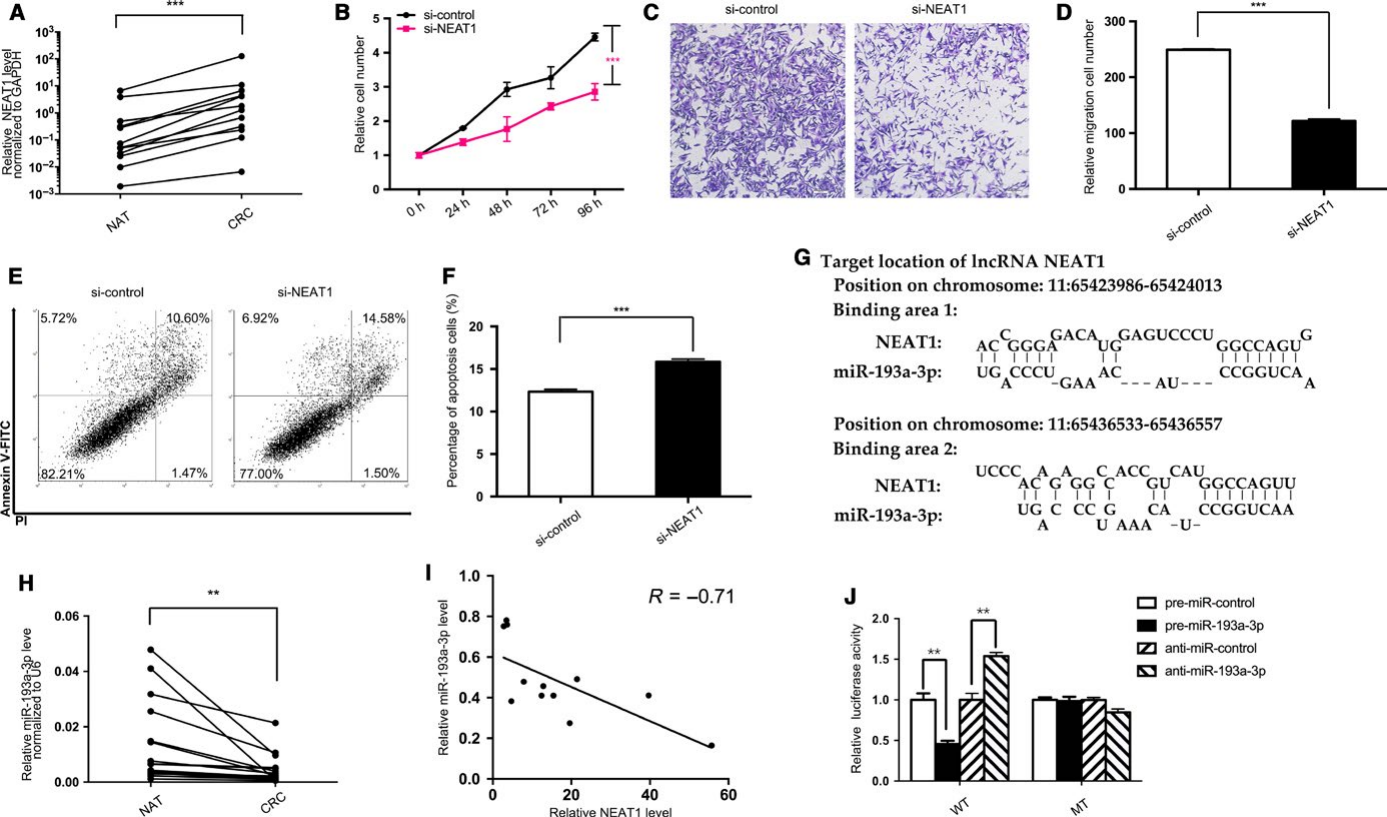
Prediction and confirmation of NEAT1/miR‐193a‐3p's binding. A, Quantitative RT‐PCR analysis of the expression levels (NEAT1 lncRNA vs GAPDH mRNA) of NEAT1 in the 12 pairs of colorectal cancer (CRC) and normal adjacent tissues (NAT). B‐F, Cell functional assays performed after the transfection of SW480 cells with equal doses of NEAT1 siRNA (si‐NEAT1) or scrambled negative control RNA (si‐control). B, Proliferation. C, Migration. D, The number of migrated cells was quantified. E, Apoptosis. F, The percentage of apoptosis cells was quantified. G, Predicted miR‐193a‐3p of seed sequences targeting NEAT1 by LncBase v2. H, Quantitative RT‐PCR analysis of the expression levels (miR‐193a‐3p vs U6) of miR‐193a‐3p in the aforementioned CRC and NAT samples. I, Pearson's correlation scatter plot of the fold change of NEAT1 and miR‐193a‐3p in CRC samples. J, Recognition of the NEAT1 seed sequences by miR‐193a‐3p. Firefly luciferase reporters containing either wild‐type (WT) or mutant (MUT) miR‐193a‐3p binding sites in NEAT1 were co‐transfected into 293T cells with equal doses of miR‐193a‐3p mimics, inhibitors, or scrambled negative control RNAs. Forty‐eight hours post‐transfection, the cells were assayed using a luciferase assay kit. The results are presented as the mean ± SD of three independent experiments. (**P < 0.01; ***P < 0.001).

To probe into the role NEAT1 plays in CRC, the expression patterns of NEAT1 content were first studied in human CRC tissues. As found, the expression level of NEAT1 increased in CRC samples in contrast with NAT samples (Figure 1A), complying with previous researches conducted on CRC tissues.^16^ To validate the vital role of NEAT1 in CRC tissue and cell line, we examined the impact exerted by artificially decreasing NEAT1 on colorectal cancer cell proliferation with NEAT1 siRNA. Two groups of CRC cell were transfected with control siRNA and NEAT1 siRNA, respectively. As expected, CRC cells transfected with NEAT1 siRNA showed the suppressed capabilities to proliferate (Figure 1B), migrate (Figure 1C,D), and the enhanced percentage of apoptosis (Figure 1E,F). Several underlying mechanisms of NEAT1 were hypothesized, and eventually, the miRNA sponges or competing endogenous RNAs was particularly stressed.^33^ As three algorithms were scanned (miRcode,^34^ LncBase v2,^35^ and starBase v2.0^36^), six miRNA families were identified (Table 1) and eventually miR‐193a‐3p was deemed as the most competitive candidate targeting NEAT1(Figure S1A,B). Two of the complementary binding sites between hsa‐miR‐193a‐3p and NEAT1 were presented in (Figure 1G). To further verify the two noncoding RNAs’ relationship, miR‐193a‐3p level was re‐examined in the previous mentioned CRC and NAT samples, whose result suggested suppressed miR‐193a‐3p content (Figure 1H), and the inverse relationship between NEAT1 and miR‐193a‐3p with the negative coefficient −0.71 (Figure 1I). Additionally, miR‐193a‐3p was reported as a target of NEAT1 through transfecting luciferase reporter plasmids of wild‐type/mutant NEAT1‐ miR‐193a‐3p binding sites to 293T cell. The group transfected with wild‐type NEAT1 and pre‐miR‐193a‐3p (mimics) took on a decreased activity lower than that with wild‐type NEAT1 and negative control. However, the group transfected with wild‐type NEAT1 and anti‐miR‐193a‐3p (inhibitors) presented an increased activity. The results were in contrast with the nonsignificant differences found in the two mutant groups (Figure 1J). Generally, these data confirmed that miR‐193a‐3p is bound by NEAT1, which complies with the existing reports and bioinformatics predictions.

**Table 1:** Nuclear paraspeckle assembly transcript 1 target candidates that have high primate conservation (Data collected form miRcode.com)

microRNA family	Seed position	Seed type	Primates conservation (%)
miR‐96/507/1271	chr11:65203169	7‐mer‐A1	89
miR‐141/200a	chr11:65201805	8‐mer	89
miR‐182	chr11:65203169	7‐mer‐A1	89
miR‐193/193b/193a‐3p	chr11:65204029	7‐mer‐m8	89
miR‐218/218a	chr11:65193974	7‐mer‐A1	89
miR‐23abc/23b‐3p	chr11:65199746	7‐mer‐A1	100

### 3.2 Knockdown NEAT1 upregulates miR‐193a‐3p expression and attenuates CRC cells

**Figure 2:**
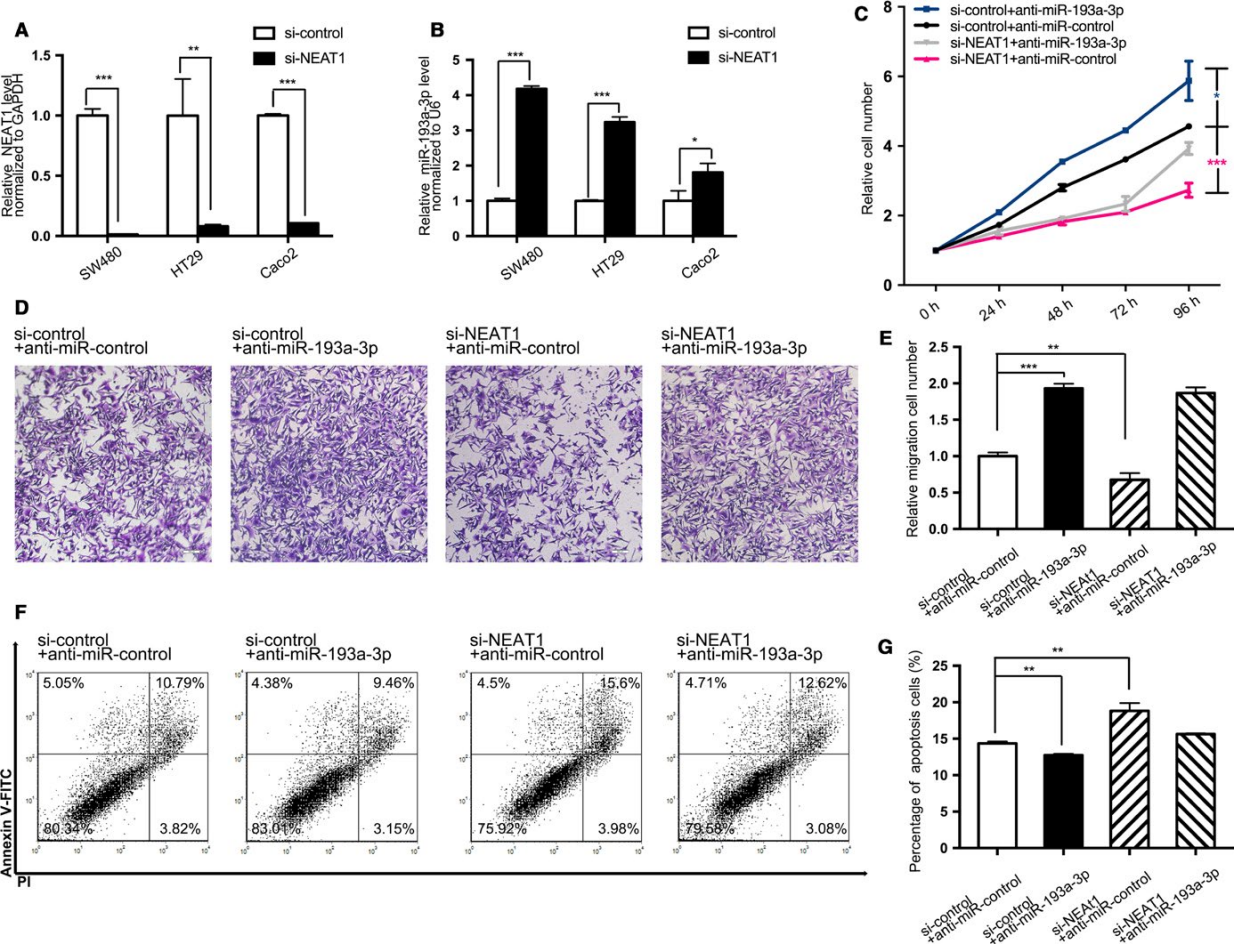
Effect of NEAT1 siRNA and anti‐miR‐193a‐3p on the CRC cell lines. A,B, Quantitative RT‐PCR analysis of the expression levels of NEAT1 and miR‐193a‐3p in SW480, HT29, and Caco2 cells transfected with equal doses of the si‐NEAT1 and si‐control. A, Expression levels of NEAT1. B, Expression levels of miR‐193a‐3p. C, The cell proliferation assay after four groups of SW480 transfected with equal doses of negative control, NEAT1 siRNA, miR‐193a‐3p inhibitors, and NEAT1 siRNA plus miR‐193a‐3p inhibitors. D, Transwell assay 48 h after aforementioned four groups’ transfection. E, The number of migrated cells was quantified. F, The apoptosis assay 48 h after aforementioned four groups’ transfection. G, The percentage of apoptosis cells was quantified. The results are presented as the mean ± SD of three independent experiments. (*P < 0.05; **P < 0.01; ***P < 0.001).

Next, this study sought to elucidate the impact exerted by NEAT1 on CRC cells through interfering NEAT1 with synthesized siRNA. Meantime, miR‐193a‐3p inhibitors were transfected to observe changes. First, SW480, HT29, and Caco2 cells were transfected with NEAT1 siRNA, and the transfection efficiency was measured by quantitative RT‐PCR analysis, as presented in Figure 2A. All three CRC cells attained adequate interfering percentage over 90%. It is noteworthy that while the NEAT1 expression had been restrained, the miR‐193a‐3p level had correspondingly increased (Figure 2B) in each cell line, which implied NEAT1's latent function in miRNA sponge or endogenous competition. To assess further impacts of interfering NEAT1, we selected SW480, with the most evident fold change in both NEAT1 and miR‐193a‐3p after interfering NEAT1, to perform a series of function experiments. Same as presented in Figure 1B, the proliferation capacity of SW480 could be confined by NEAT1 siRNA. Yet such impact was counteracted through transfecting miR‐193a‐3p inhibitors in 72 hours (Figure 2C). Also, SW480's capabilities to migrate were queried by Transwell migration assay, and poorer migration competency arose from NEAT1 deficiency, whereas was rescued by miR‐193a‐3p inhibitors in 48 hours (Figure 2D,E). Lastly, apoptosis was inspected in SW480 with flow cytometric analysis. A larger percentage of apoptotic cells were presented in the cells transfected with NEAT1 siRNA than control group. Yet as anticipated, miR‐193a‐3p inhibitors decreased apoptosis rate and weakened the effect made by NEAT1 siRNA (Figure 2F,G). As indicated in all these assays, the decreased NEAT1 impaired the CRC cell's viability, whereas could be offset by miR‐193a‐3p inhibitors, which could intensify CRC cells' proliferation and migration but reduce apoptosis.

### 3.3 KRAS acts as a target of miR‐193a‐3p

**Figure 3:**
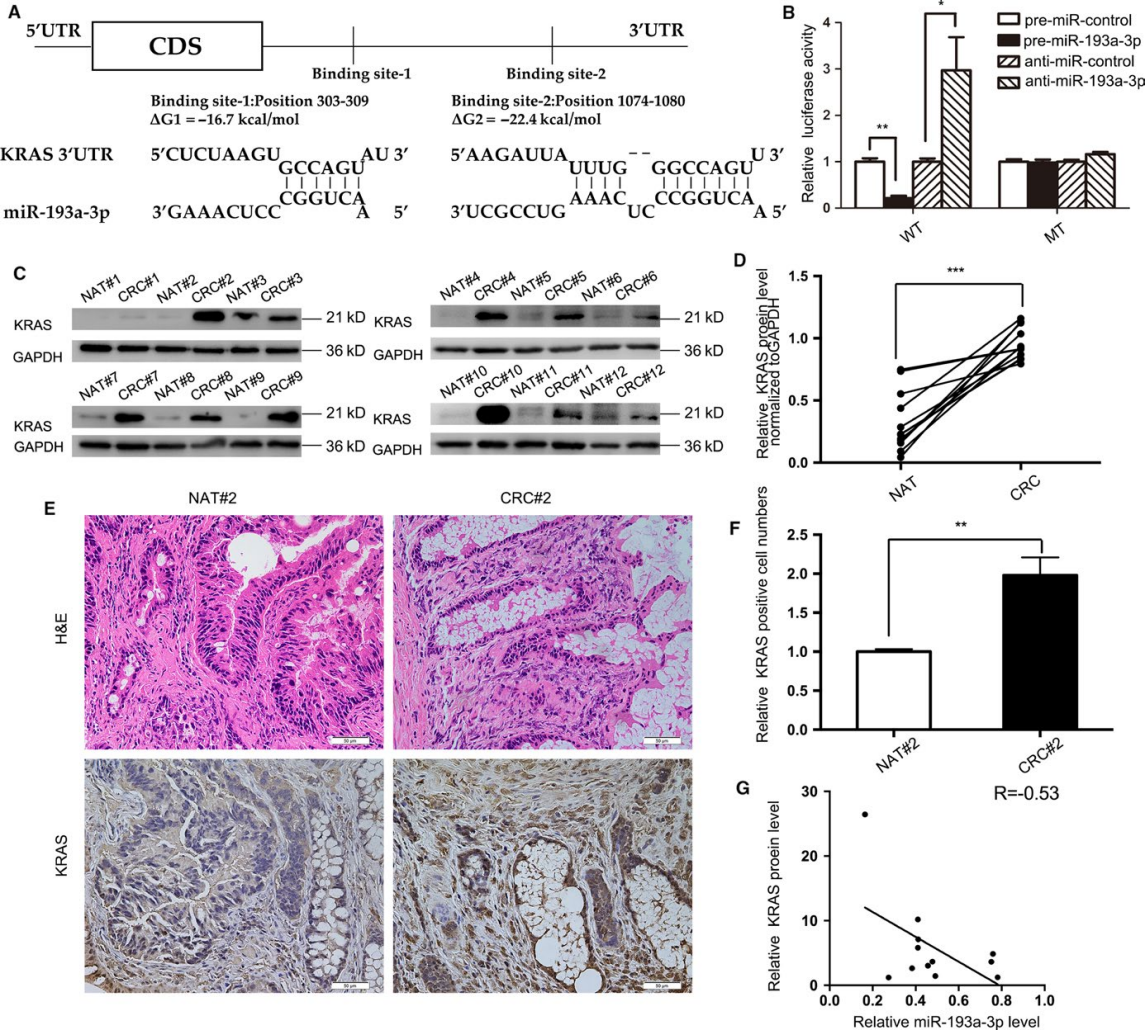
Prediction and confirmation of miR‐193a‐3p/KRAS's binding. A, Predicted binding sites in KRAS 3′‐UTR and miR‐193a‐3p, with free energy values of each hybrid indicated. B, Recognition of the KRAS 3′‐UTR by miR‐193a‐3p. Firefly luciferase reporters containing either wild‐type (WT) or mutant (MUT) miR‐193a‐3p binding sites in the KRAS 3′‐UTR were co‐transfected into 293T cells with equal doses of the miR‐193a‐3p mimics, inhibitors and negative control. Forty‐eight hours post‐transfection, the cells were assayed using a luciferase assay kit. C,D, Western blotting and quantitative analysis (KRAS protein vs GAPDH protein) of the expression levels of the KRAS protein in aforementioned 12 pairs of CRC and NAT samples. E,F, H&E‐stained sections and immunohistochemical staining for KRAS in the one pair of aforementioned CRC samples followed by quantitative analysis for KRAS‐positive cells. G, Pearson's correlation scatter plot of the fold change of miR‐193a‐3p and KRAS in the aforementioned specimens. The results are presented as the mean ± SD of three independent experiments. (*P < 0.05; **P < 0.01; ***P < 0.001).

Given that miR‐193a‐3p inhibitor is a CRC promoter and miRNAs' posttranscriptional control has suggested by recent studies in targeting mRNA,^20^ this study planned to further determine the role of miR‐193a‐3p played in CRC. This study hypothesized that miR‐193a‐3p in CRC works as a tumor suppressor, involving miRNA target pairs. As three computational algorithms were scanned (PincTar21,^37^ miRanda8,^38^ and TargetScan20^37^), SLC10A6, FIL1, ERBB4, KRAS, MMP19, etc, were predicted as candidates for the downstream targets of miR‐193a‐3p. Meantime, we searched The Cancer Genome Atlas (TCGA) database to screen the possible target for colorectal cancer. Finally, KRAS, kirsten rat sarcoma viral oncogene homolog, which is presented as the fourth most frequently affected gene in colorectal cancer (231/537 [43.02%]) (Figure S2) and overlaps aforementioned search results, was chosen for next validation. Two predicted hybridizations between miR‐193a‐3p and the 3'UTR of KRAS are presented in Figure 3A. The two hybridizations' minimum free energy values are −16.7 and −22.4 kcal/mol, respectively, both complying with the range of genuine miRNA target pairs. Specifically, to verify their binding, the target sequence of KRAS 3′‐UTR was cloned into a luciferase reporter plasmid. A decrease was observed in luciferase reporter activity in the 293T cell treated with miR‐193a‐3p mimics and target KRAS sequence while an increase in the miR‐193a‐3p inhibitors and KRAS, in contrast to another two groups with mutant KRAS sequence and no statistical difference in reporter activity (Figure 3B).

Furthermore, 12 pairs of CRC tissues and NAT adopted in Figure 1 were re‐measured for KRAS protein. KRAS protein expression levels were evidently higher in CRC specimens than those in NAT, as demonstrated in Figure 3C,D. Immunohistochemical staining also manifested the presence of higher KRAS in one of the pairs of tissues (Figure 3E,F). Finally, the negative correlation between miR‐193a‐3p and KRAS was calculated and illustrated in Figure 3G.

### 3.4 miR‐193a‐3p suppresses CRC cell biological behaviors by controlling KRAS expression

**Figure 4:**
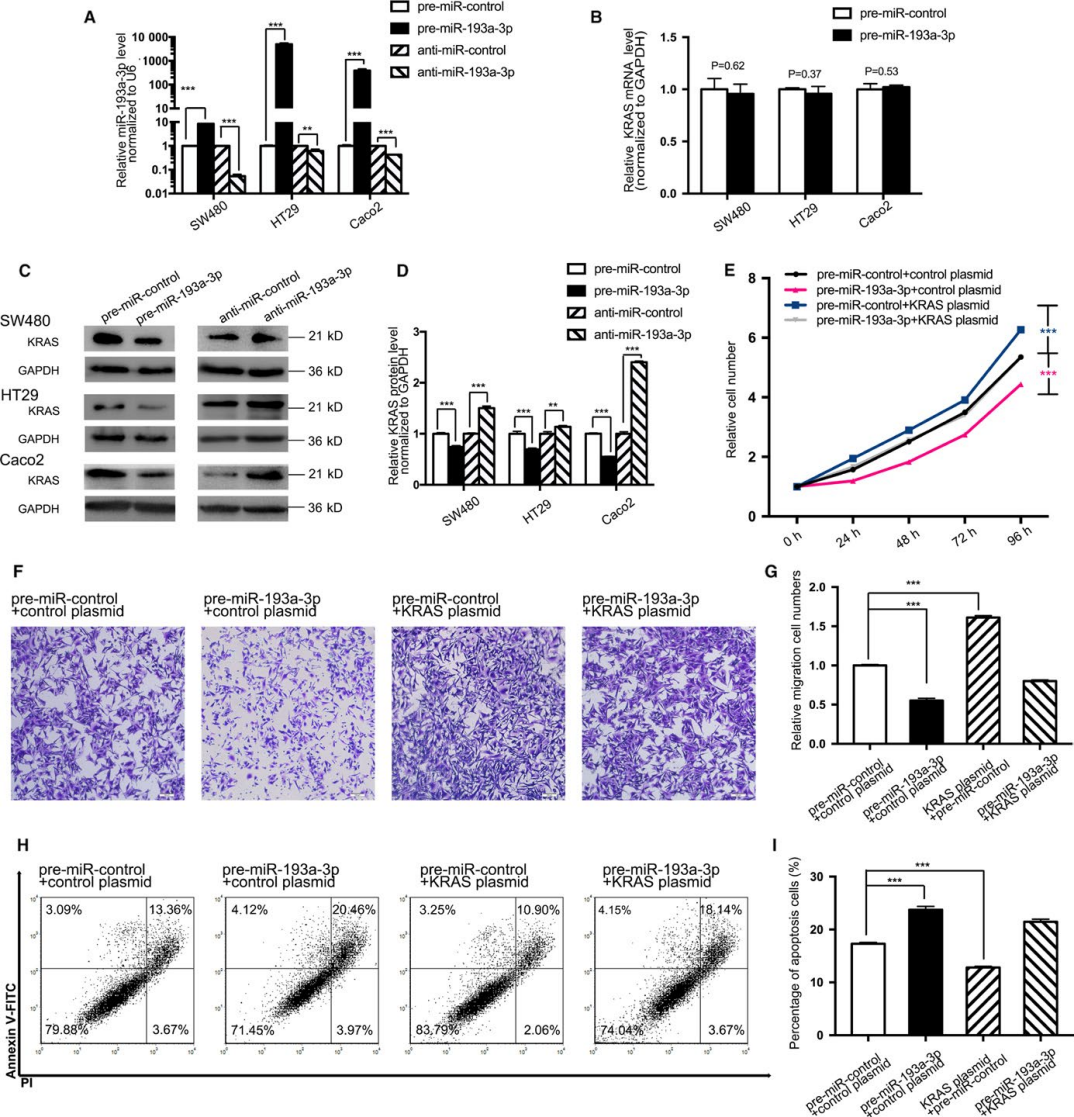
Effect of miR‐193a‐3p and KRAS plasmid on the CRC cell lines. A, Quantitative RT‐PCR analysis of the expression levels of miR‐193a‐3p in SW480, HT29, and Caco2 cells transfected with equal doses of miR‐193a‐3p mimics, inhibitors, and negative control. B, Quantitative RT‐PCR analysis of the expression levels of KRAS mRNA in SW480, HT29, and Caco2 cells transfected with equal doses of miR‐193a‐3p mimics, inhibitors, and negative control. C,D, Western blotting and the quantitative analysis of KRAS protein levels in SW480, HT29, and Caco2 cells after transfected with equal doses of the miR‐193a‐3p mimics, inhibitors, and negative control. E, The cell proliferation assay after four groups of SW480 transfected with equal doses of negative control, KRAS plasmid, miR‐193a‐3p mimics, and KRAS plasmid plus miR‐193a‐3p mimics. F,G, Transwell assay 48 h after aforementioned four groups’ transfection. H,I, The apoptosis assay 48 h after aforementioned four groups’ transfection. The results are presented as the mean ± SD of three independent experiments. (**P < 0.01; ***P < 0.001).

Then, miR‐193a‐3p and KRAS protein expression in three CRC cells (SW480, HT29, and Caco2) was evaluated after overexpression or knockdown of miR‐193a‐3p (Figure 4A,C,D). KRAS protein expression in all three CRC cells had decreased by over 50% after miR‐193a‐3p mimics transfection, whereas increased with miR‐193a‐3p inhibitors treatment. Furthermore, to confirm the level at which miR‐193a‐3p modulates KRAS protein, KRAS mRNA was also quantified as miR‐193a‐3p mimics were transfected (Figure 4B). None of the CRC cells took on statistical disparity on mRNA level, which is also supported by miRNA's mRNA posttranscriptional control theory.

Similarly, the proliferation, migration, and apoptosis assays were projected to assess miR‐193a‐3p and KRAS's impact on SW480. An expression plasmid was designed to specifically express the full‐length KRAS ORF without the miR‐193a‐3p‐responsive 3'‐UTR. SW480 transfected with miR‐193a‐3p mimics showed decreased proliferation (Figure 4E), migration (Figure 4F,G), and enhanced percentage of apoptotic cells (Figure 4H,I). However, KRAS plasmid could not only advance proliferation and migration as well as inhibit apoptosis, but also weaken the effects exerted by miR‐193a‐3p on the cells (Figure 4E‐I). In conclusion nutshell, the results here jointly substantiated miR‐193a‐3p's binding to the 3′‐UTR of KRAS transcript and the miRNA's posttranscriptional control.

### 3.5 Knockdown NEAT1 circuitously modulate KRAS expression through miR‐193a‐3p both in vitro and in vivo

**Figure 5:**
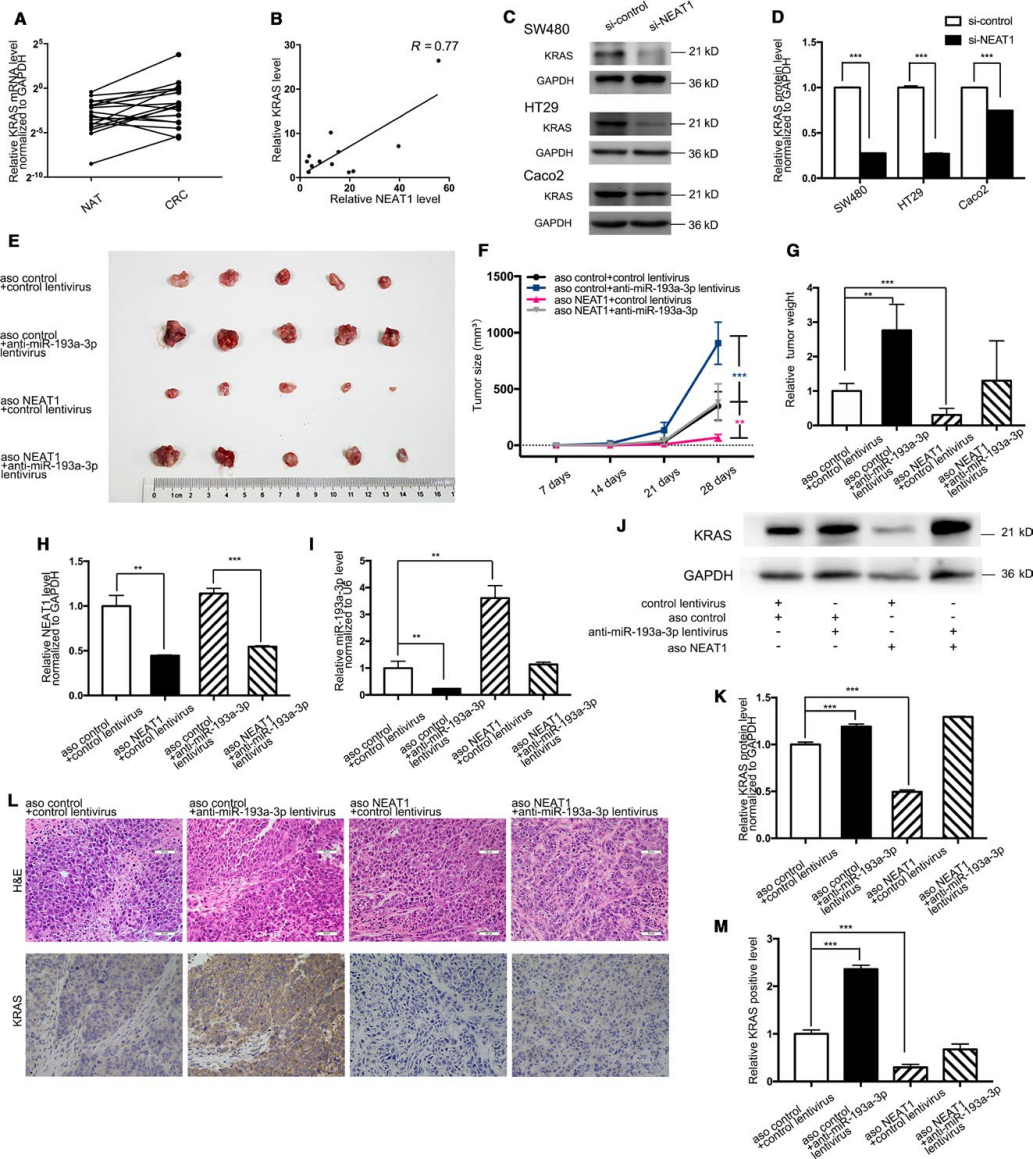
Effect of si‐NEAT1/aso NEAT1 and anti‐miR‐193a‐3p on KRAS and the development of CRC cell as well as xenografts in nude mice. A, Quantitative RT‐PCR analysis of the expression levels (KRAS mRNA vs GAPDH mRNA) of KRAS in the aforementioned CRC and NAT. B, Pearson's correlation scatter plot of the fold change of NEAT1 and KRAS in CRC samples. C,D, Western blotting analysis and quantitative comparison of KRAS protein levels in SW480, HT29, and Caco2 cells after transfected with equal doses of the si‐NEAT1 or si‐control. E, SW480 cells co‐infected with negative control, aso NEAT1, miR‐193a‐3p inhibitor lentivirus, and aso NEAT1 plus miR‐193a‐3p inhibitor lentivirus were transplanted subcutaneously into nude mice and harvested. F, Tumor size of subcutaneous implantation models of SW480 cells. G, Quantitative comparison of tumor weight between the four groups. H, Quantitative RT‐PCR analysis of the expression levels (NEAT1 lncRNA vs GAPDH mRNA) of NEAT1 in the tumors from the four groups. I, Quantitative RT‐PCR analysis of the expression levels (miR‐193a‐3p vs U6) of miR‐193a‐3p in the tumors from the four groups. J,K, Western blotting and quantitative analysis of KRAS protein levels in the tumors from the four groups. L, H&E‐stained sections and immunohistochemical staining for KRAS in the tumors from the four groups. M, Quantitative comparison of KRAS‐positive cells between the four groups. The results are presented as the mean ± SD of three independent experiments. (**P < 0.01; ***P < 0.001).

To incorporate the upstream with downstream, KRAS mRNA content of 12 pairs of CRC & NAT was reappraised, whereas no difference was found of statistical significance between the two groups (Figure 5A). Then, the correlation between NEAT1 and KRAS protein level in these tissues was analyzed, and the positive corresponding relation was acquired with coefficient 0.77 (Figure 5B). Also, as indicated by western blotting, KRAS protein would decrease after CRC cells (SW480, HT29, and Caco2) transfected with NEAT1 siRNA (Figure 5C,D), whereas without any mRNA alteration (not presented in data).

On that basis, a CRC xenograft mouse model was employed to assess the further impact of NEAT1, miR‐193a‐3p, and KRAS. Four groups of SW480 cells (2 × 10^6^ cells per 0.1 mL) were co‐infected, respectively, with the negative control, aso NEAT1, miR‐193a‐3p inhibitor lentiviral expression plasmid, aso NEAT1 plus miR‐193a‐3p inhibitor lentiviral plasmid. The cells were implanted subcutaneously into 5‐week‐old nude mice. After 28‐day xenograft growth in vivo, the mice were sacrificed (Figure 5E). The aso NEAT1 group tumors’ size and weight evidently decreased compared to those in the control group. Yet tumor size and weight discovered in miR‐193a‐3p inhibitor group conspicuously increased. Besides, miR‐193a‐3p inhibitor overexpression weakened the suppressive impact exerted by aso NEAT1(Figure 5F,G). Also, overall RNA and proteins were isolated from the tumors and then analyzed. With adequate knockdown of NEAT1 (Figure 5H), increase in miR‐193a‐3p (Figure 5I), and nonsignificant contrast in KRAS mRNA (not presented in data), KRAS protein expression varied with each group. Tumors with aso NEAT1 took on less KRAS protein, whereas the miR‐193a‐3p inhibitor group displayed higher KRAS and rescued the suppression exerted by aso NEAT1 in the last group (Figure 5J,K). Additionally, hematoxylin and eosin (H&E) staining of xenograft tissues manifests more cell mitosis areas in aso NEAT1 group. Xenografts co‐infected with both aso NEAT1 and miR‐193a‐3p inhibitor lentiviral plasmid took on alleviated cell mitosis compared to aso NEAT1 group (Figure 5L). Immunohistochemical staining uncovered the presence of lower levels of KRAS in the tumors from aso NEAT1 group, but the reversed consequence in miR‐193a‐3p inhibitor group. In the last group, miR‐193a‐3p inhibitor's oncogenic function rescued the number of cells stained with KRAS antibody (Fig5L,M). These results all complied with the results in vitro assays, strengthening the previous confirmation.

**Figure 6:**
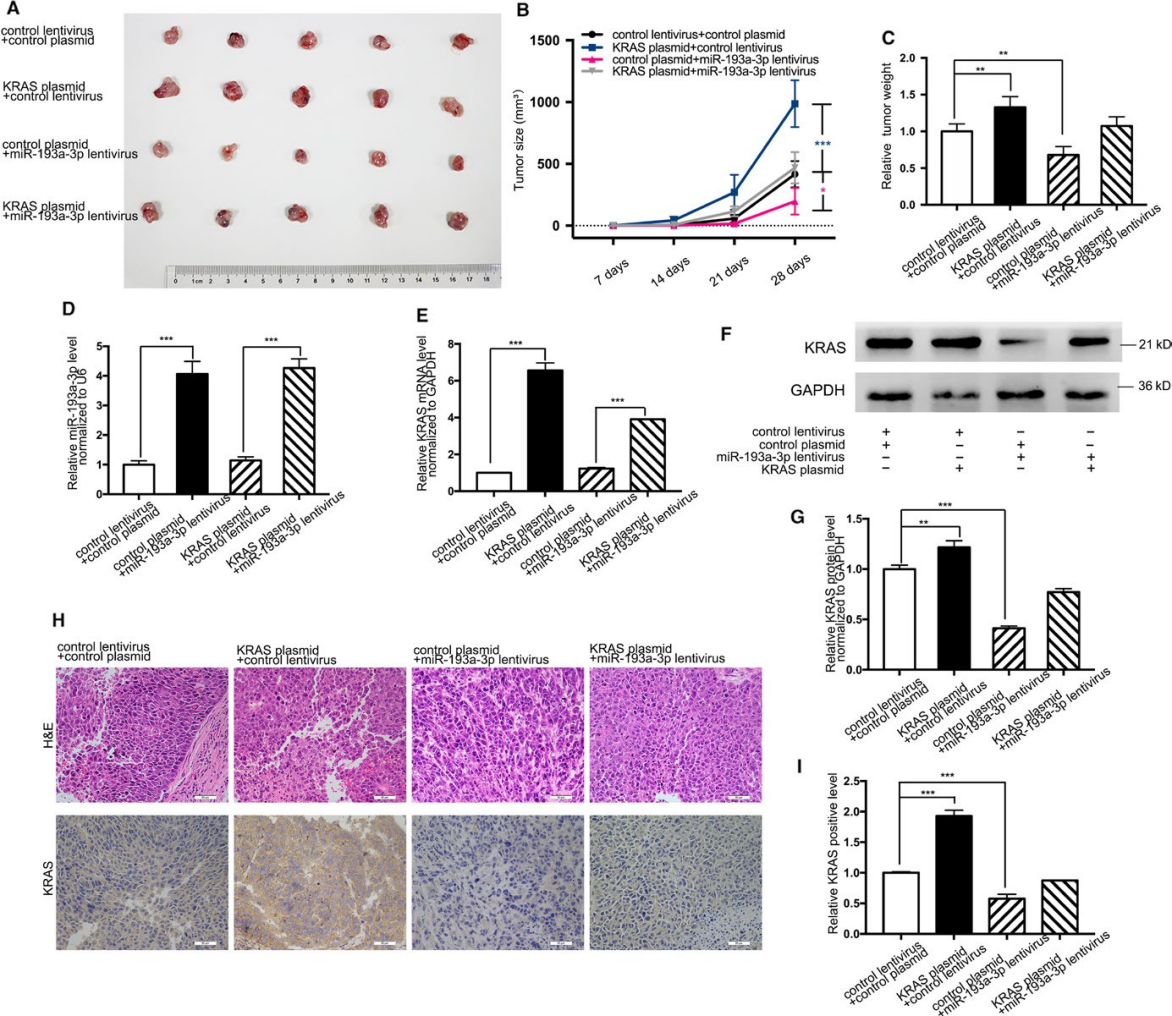
Effect of miR‐193a‐3p and KRAS plasmid on the development of CRC cell xenografts in nude mice. A, SW480 cells co‐infected with negative control, KRAS plasmid, miR‐193a‐3p mimic lentivirus, KRAS plasmid plus miR‐193a‐3p mimic lentivirus were transplanted subcutaneously into nude mice and harvested. B, Tumor size of subcutaneous implantation models of SW480 cells. C, Quantitative comparison of tumor weight between the four groups. D, Quantitative RT‐PCR analysis of the expression levels (miR‐193a‐3p vs U6) of miR‐193a‐3p in the tumors from the four groups. E, Quantitative RT‐PCR analysis of the expression levels (KRAS mRNA vs GAPDH mRNA) of KRAS in the tumors from the four groups. F,G, Western blotting and quantitative analysis of KRAS protein levels in the tumors from the four groups. H, H&E‐stained sections and immunohistochemical staining for KRAS in the tumors from the four groups. I, Quantitative comparison of KRAS‐positive cells between the four groups. The results are presented as the mean ± SD of three independent experiments. (*P < 0.05; **P < 0.01; ***P < 0.001).

Likewise, downstream control was reconfirmed with a CRC xenograft mouse model. Four groups of 5‐week‐old nude mice were subcutaneously transplanted with SW480 cells (transfected with negative control, KRAS plasmid, miR‐193a‐3p mimic lentivirus, and the mixture of above two, respectively). Similar methods were applied to verify the downstream modulation, including tumor weight and size (Figure 6A‐C), quantitative RT‐PCR examination of miR‐193a‐3p and KRAS mRNA (Figure 6D,E), western blotting of KRAS protein level (Figure 6F,G), and H&E/Immunohistochemical staining (Figure 6H,I) after 28‐day xenograft growth in the mice. These consequences verified miR‐193a‐3p as a tumor suppressor, whose impact could be reversed by oncogenic KRAS, which further verified the preceding validation of miR‐193a‐3p/KRAS control in this article.

**Figure 7:**
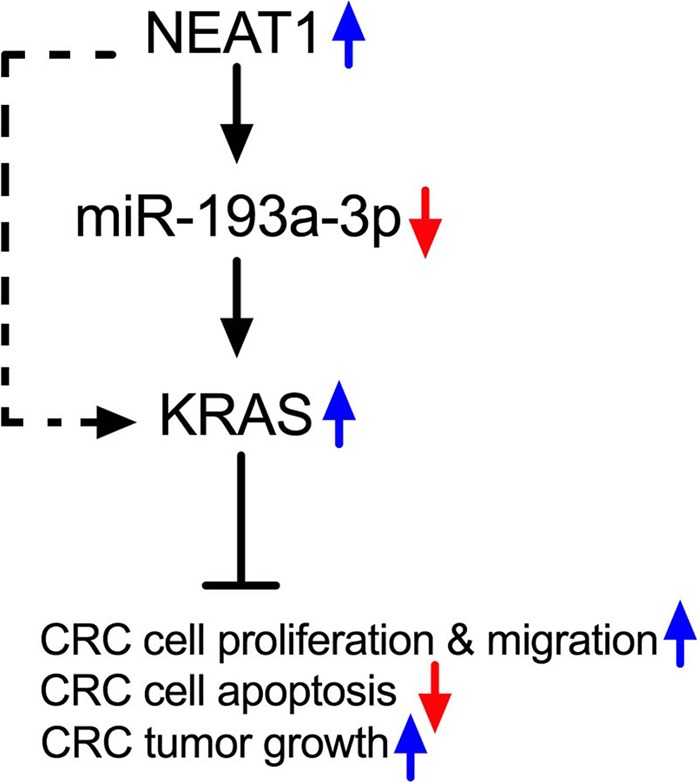
Working model of the NEAT1/miR‐193a‐3p/KRAS axis in CRC

Taken together, this study uncovered an essential NEAT1/miR‐193a‐3p/KRAS regulatory axis in colorectal cancer. The working model is summarized in Figure 7.

## 4 DISCUSSION

In summary nutshell, our findings on CRC tissues, cell lines, and xenograft models overall support that NEAT1 serves as an oncogene, with knockdown note worthily attenuating CRC cell development. The possible mechanism of interactions between NEAT1 and the most competitive candidate miR‐193a‐3p, predicted by bioinformatics algorithms, is validated initially by our team. As verified here, when NEAT1 was silenced, the CRC cells’ viability could be oppressed, either via small interfering RNAs or antisense oligonucleotides. Yet knockdown of miR‐193a‐3p could reverse the impact exerted by NEAT1 siRNA through controlling KRAS. That's might be the reason why knockdown of NEAT1 or increase of miR‐193a‐3p could impede cancer progression in colorectal carcinogenesis and offer genetic evidence that NEAT1 is one of the critical regulators to control oncogene KRAS.

The oncogenic implications of NEAT1 on diversified cancers have been proved in previous relevant studies. Originally, we were confused with the expression level and clinicopathological phenomena in clinical cancer samples. As having been reported, NEAT1 takes on higher content and relates to tumor recurrence and unfavorable prognosis in CRC.^16^, ^39^ Gastric adenocarcinoma,^40^ hepatocellular carcinoma,^41^ and breast cancers,^42^ etc, were either ascertained increased NEAT1 amount in cancer samples compared to the noncancerous ones. Moreover, increase of NEAT1 was incorporated with deteriorated clinicopathological parameters, for example multi‐tumor nodes, metastasis, portal vein tumor embolus, vaso‐invasion, and tumor capsule infiltration,^41^ which implies additional functions of NEAT1 to administrate tumor genesis and developments. In general, most researches concerning NEAT1 oncogenic mechanism applied NEAT1 RNA interference or other gene silencing techniques, including but not limited to short hairpin (sh) RNA and antisense oligonucleotides. For instance, NEAT1’s other targets, miR‐129/CTBP2,^43^ miR‐335‐3p/c‐met,^44^ miR‐107/CDK6,^45^ miR‐194,^46^ and hnRNP A2^47^were verified through interrupting NEAT1; meantime, Oct4 was proved as an upstream of NEAT1^48^ with the depletion of NEAT1. It could be achieved to inhibit cancer cell progression through disrupting NEAT1, and impaired capacity of proliferation, migration, invasion, and enhanced apoptosis was observed in assorted cancer cell lines. All foregoing researches aroused our curiosity on CRC cells with the interference of NEAT1. It was also noticed that NEAT1 stable overexpression has been adopted in recent researches as technologies in nucleus leap advanced. Not only NEAT1’s other predicted targets miR‐337‐3p/E2F3,^49^ miR‐34/BCL2,^50^ but also the estrogen receptor alpha,^51^ a NEAT1 regulator, were confirmed with the overexpressed sequence of NEAT1. In this regard, oncogenic functions of NEAT1 were consolidated, and the entangled mechanisms were elucidated to a greater extent. Yet some investigations manifested conflicting consequence, defining NEAT1 as a tumor suppressor. As found in nasopharyngeal carcinoma, NEAT1 checked cancer development through miR‐101‐3p.^52^ In pancreatic cancer, NEAT1 was a p53‐inducible lncRNA essential to suppress transformation.^53^ The paradoxical outcome mirrors labyrinthine controlling networks of NEAT1 and provokes our greater interest in NEAT1.

Despite the ambiguous definition of NEAT1 in various investigations, miRNAs and its posttranscriptional control in cancer have been unequivocally published for years. Dysregulations of miRNA expression not only reveal the mechanism in tumor progression but also emerge as a potential therapeutic strategy. miR‐193a‐3p, which is reported to decrease in lung cancer, hepatocellular carcinoma, colorectal cancer, ovarian cancer, and pleural mesothelioma, etc,^27^, ^30^, ^32^, ^54^, ^55^ controls varied proteins post‐transcriptionally and serves as a tumor suppressor that could be potentially useful as therapeutic molecules against cancer development. The tumor suppressive mechanism of miR‐193a‐3p has been expounded by GRB7 and MAPK/ERK pathways,^32^ cyclin D1,^56^ PTEN,^57^ AJUBA,^58^ etc in varied cancers. Here, we selected KRAS, which is one essential component of Ras family and plays a crucial role in CRC, as miR‐193a‐3p's target. Oncogenic activation of KRAS occurs in approximately 40% of all CRCs. Aberrant KRAS activation leads to cascade effects of RAS/RAF/MAPK and PI3K/AKT/mTOR pathways, which further contribute to the formation of CRC. This study eventually elucidates where KRAS situates in the noncoding RNA‐related pathway and the mechanism of two regulators of KRAS in CRC. Taken together, the correlation between NEAT1 and KRAS was ultimately confirmed. Our research provided the evidence that targeting NEAT1 could reduce the content of KRAS, as well as suppress CRC cells either in vitro or in vivo, which might supply novel target for future CRC therapy.

It's worth mentioning that besides regulating miRNA activity, lncRNA has following other methods to affect gene expression: (a) lncRNA could regulate mRNA splicing and maturation. For instance, lncRNA MALAT1 was reported to correlate with serine/arginine splicing factors in the nuclear speckles.^59^ lncRNA 5S‐OT regulates alternative splicing multiple genes after an antisense Alu element was inserted.^60^ (b) lncRNA could control nuclear/cytoplasm shuttling of mRNA and thus affects mRNA's translation and results protein level's change. (c) lncRNA could control mRNA and protein stability. (d) lncRNA may lead to dicer‐dependent endogenous siRNA production.^61^ It is still possible that NEAT1 influenced KRAS protein level through above‐mentioned ways. Additionally, other miRNA candidates (including but not limited to miR‐140‐5p, miR‐218, miR‐23b‐3p, etc) may also have potential targets to regulate KRAS.

Also, this research can be further optimized. First, as stated above, other than the interaction between lncRNA‐miRNA‐mRNA, other lncRNA pathways should be considered. We would like to further our research on NEAT1’s potential that could directly influence stability of mRNA or proteins. For example, NEAT1 was reported to be upregulated by proteasomal inhibition and in turn to protect fibroblasts from cell death triggered by proteasome inhibition.^62^ Second, the overexpression of NEAT1 either via lentivirus or nucleofection, as well as the NEAT1 gene knockout mice, could be applied to continue this study. Third, the screen process could not be limited to silico tools. Imperfect seed match between RNAs or targeting sites outside of 3’UTR could exist, which leads to false positive predictions.^63^ Biotinylated‐RNA pulldown as well as RNA overexpression, combined with RNA sequencing, could be more accurate and effective. Subsequent experiments are being prepared, as expected to further expound mechanism and provide more exact treatment in CRC.

## 5 ETHICS STATEMENT

The research was approved by the Ethics Committee of Nanjing Drum Tower Hospital, the Affiliated Hospital of Nanjing University Medical School. Written informed consent was obtained from all participants.

## CONFLICT OF INTEREST

There is no conflict of interests.
