# Supplementary figures and images for: Knockdown long noncoding RNA nuclear paraspeckle assembly transcript 1 suppresses colorectal cancer through modulating miR‐193a‐3p/KRAS
Source: Cancer Med. 2018 Dec 21;8(1):261–75. doi: 10.1002/cam4.1798 (PMC6346262; doi:10.1002/cam4.1798)

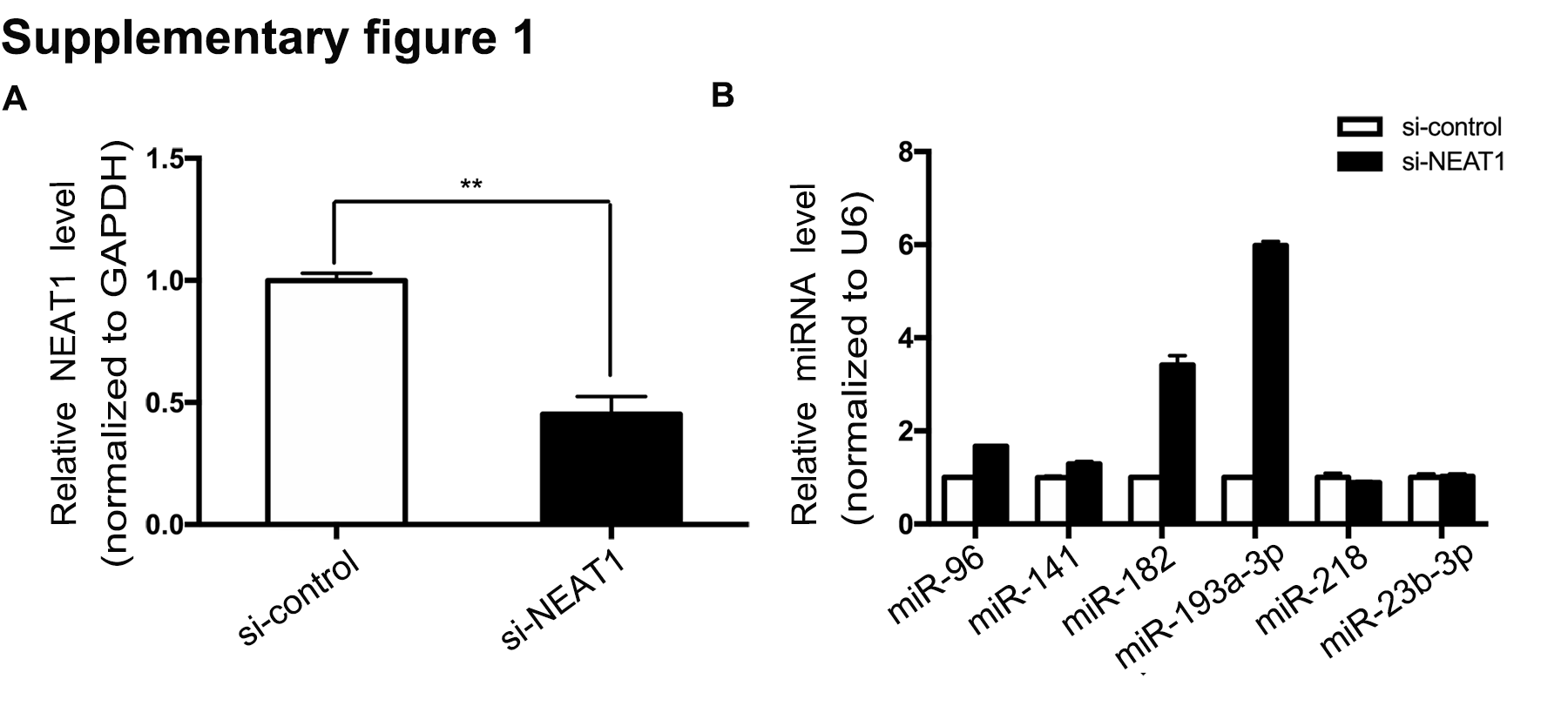

Supplement: Supplementary file 1 [file CAM4-8-261-s001.tif]

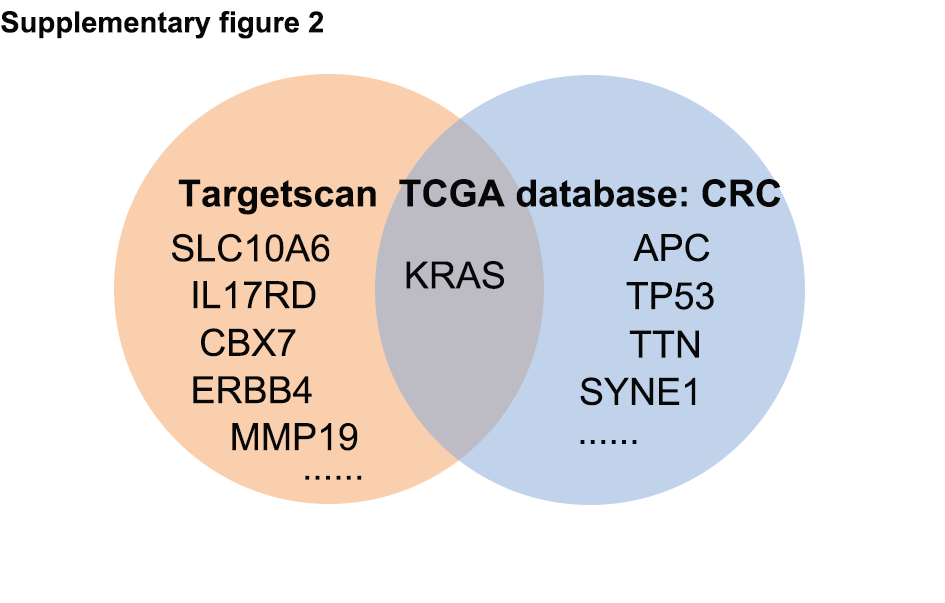

Supplement: Supplementary file 2 [file CAM4-8-261-s002.tif]
